# Calcium imaging in intact mouse acinar cells in acute pancreas tissue slices

**DOI:** 10.1371/journal.pone.0268644

**Published:** 2022-06-03

**Authors:** Urška Marolt, Eva Paradiž Leitgeb, Viljem Pohorec, Saška Lipovšek, Viktória Venglovecz, Eleonóra Gál, Attila Ébert, István Menyhárt, Stojan Potrč, Marko Gosak, Jurij Dolenšek, Andraž Stožer

**Affiliations:** 1 Clinical department for abdominal and general surgery, University Medical Centre Maribor, Maribor, Slovenia; 2 Institute of Physiology, Faculty of Medicine, University of Maribor, Maribor, Slovenia; 3 Faculty of Natural Sciences and Mathematics, University of Maribor, Maribor, Slovenia; 4 Faculty of Chemistry and Chemical Engineering, University of Maribor, Maribor, Slovenia; 5 Department of Pharmacology and Pharmacotherapy, University of Szeged, Szeged, Hungary; Vrije Universiteit Brussel, BELGIUM

## Abstract

The physiology and pathophysiology of the exocrine pancreas are in close connection to changes in intra-cellular Ca^2+^ concentration. Most of our knowledge is based on *in vitro* experiments on acinar cells or acini enzymatically isolated from their surroundings, which can alter their structure, physiology, and limit our understanding. Due to these limitations, the acute pancreas tissue slice technique was introduced almost two decades ago as a complementary approach to assess the morphology and physiology of both the endocrine and exocrine pancreas in a more conserved *in situ* setting. In this study, we extend previous work to functional multicellular calcium imaging on acinar cells in tissue slices. The viability and morphological characteristics of acinar cells within the tissue slice were assessed using the LIVE/DEAD assay, transmission electron microscopy, and immunofluorescence imaging. The main aim of our study was to characterize the responses of acinar cells to stimulation with acetylcholine and compare them with responses to cerulein in pancreatic tissue slices, with special emphasis on inter-cellular and inter-acinar heterogeneity and coupling. To this end, calcium imaging was performed employing confocal microscopy during stimulation with a wide range of acetylcholine concentrations and selected concentrations of cerulein. We show that various calcium oscillation parameters depend monotonically on the stimulus concentration and that the activity is rather well synchronized within acini, but not between acini. The acute pancreas tissue slice represents a viable and reliable experimental approach for the evaluation of both intra- and inter-cellular signaling characteristics of acinar cell calcium dynamics. It can be utilized to assess many cells simultaneously with a high spatiotemporal resolution, thus providing an efficient and high-yield platform for future studies of normal acinar cell biology, pathophysiology, and screening pharmacological substances.

## Introduction

With increasing numbers of patients suffering from diabetes mellitus, pancreatitis, and pancreatic cancer, understanding normal and pathological pancreas physiology is becoming more and more important [1–4]. The treatment options for these diseases are limited and they are all characterized by shorter life expectancy, poorer quality of life, and higher morbidity after treatment attempts [5, 6]. The pancreas is a bifunctional gland consisting of exocrine acini and ducts and endocrine islets of Langerhans [7]. The former regulates the digestive breakdown of energy-rich polymers in food, and the latter coordinates the postprandial storage and use of energy-rich nutrients, as well as their controlled interprandial release from internal stores [8–11]. The exocrine pancreas represents more than 90% of total organ volume and comprises acinar and ductal cells that secrete enzymes, their precursors (zymogens), and a bicarbonate-rich fluid. The remaining < 10% of the organ are endocrine cells (1–4%) and interstitial or mesenchymal components: the blood and lymphatic vessels, nerves, fibrous tissue, and immune cells [12–19]. The pancreatic exocrine function is stimulated by neurotransmitters and hormonal secretagogues, for instance, acetylcholine (ACh) and cholecystokinin (CCK). These two molecules exert their effects through different intra-cellular mediators, which liberate Ca^2+^ from intra-cellular stores, the endoplasmic reticulum (ER), and acid Ca^2+^ pools into the cytosol [20, 21]. Under physiological conditions, the rise of intra-cellular Ca^2+^ concentration ([Ca^2+^]_i_) is mainly confined to the apical region in the form of short-lasting and repetitive local [Ca^2+^]_i_ signals responsible for exocytosis and acinar fluid secretion. Eventually, the secretagogue stimulus is sufficient to raise [Ca^2+^]_i_ concentration globally towards the basal cell pole, which influences ion transport, protein synthesis, and cell metabolism [22–25]. [Ca^2+^]_i_ oscillations propagate between cells via gap junctions (Cx24, Cx32) and are believed to be synchronized among cells in the same acinus [25–30]. Sustained elevation in [Ca^2+^]_i_ concentration evoked by pathological agents, such as bile acids, fatty acids, and non-oxidative alcohol metabolites (fatty acid ethyl esters), leads to a bioenergetic collapse of the acinar cell, resulting in inappropriate intra-cellular trypsin activation, activation of nuclear factor-ҡB, cytoskeletal damage, mitochondrial dysfunction, vacuolization, and necrosis, causing cell injury and acute pancreatitis [31–35].

The key research endeavors to understand the intra-cellular mechanisms responsible for acinar cell enzyme synthesis and secretion were performed on cell lines, freshly isolated acinar cells, and isolated pancreatic acini [36, 37]. Isolation protocols employed in these methods involve digestion by enzymes, a procedure that leads to structural and functional changes, manifested as loss of microvilli and surface receptors, changes in membrane potential, upregulation of cytokines and chemokines, protein kinase activation, and reduced ACh-stimulated secretion of zymogene granules [38–40]. Furthermore, cell-to-cell contacts, which are crucial for normal pancreatic exocrine function, become interrupted during isolation of single cells [30, 41–45]. Moreover, isolation of acini does not enable paracrine-endocrine interactions, has been demonstrated to prevent contacts with the extracellular matrix and is associated with acinar cell transdifferentiation, which, taken together, ultimately alters their physiology [38, 44, 46–53]. To overcome at least some of the above stated drawbacks and enable the investigation of exo- and endocrine cells in a more natural environment, in analogy with successful similar approaches in other tissues [54–57], the acute pancreas tissue slice technique was introduced in 2003 [19, 39, 50, 58, 59]. The main advantage of this approach is the short preparation time without the need for overnight culturing, with the first slices being available for experiments in less than an hour [50, 60, 61]. The preparation involves minimum mechanical stress, no exogenous enzymatic degradation, the morphological and physiological features are preserved, and with some adaptations, the slices can be used for long periods of time [59]. The tissue slices were initially employed for *in situ* studies of endocrine beta and alpha cells [58, 62–64], and later also for acinar and ductal cells [50, 65–67] and other types of cells in the pancreas [58, 59, 65, 68–72].

With regard to [Ca^2+^]_i_ signaling in acinar cells, there is an open question as to whether the [Ca^2+^]_i_ elevations elicited by ACh and other secretagogues in isolated cells and clusters occur in undissociated preparations, and if they do, what are their properties [35]. To the best of our knowledge, it has not been possible to monitor [Ca^2+^]_i_ changes in acini *in vivo* so far [35]. One study demonstrated [Ca^2+^]_i_ oscillations in intact isolated lobuli [73] and three previous studies in acute pancreas tissue slices mention acinar cell [Ca^2+^]_i_ dynamics, two in mouse [59, 74] and one in human tissue [66]. However, these studies did not aim to quantify the responses to different levels of stimulation systematically or to assess the degree of intra- and inter-acinar heterogeneity and coupling on a large number of cells. Additionally, the spatiotemporal resolution was typically too low to enable single-cell resolution and reliable determination of oscillation properties [66]. In the present study, we therefore specifically aimed at filling this gap in our knowledge and set out to analyze the responses of acinar cells to stimulation with a wide range of concentrations of the physiological agonist ACh, with particular emphasis on the coding properties of these responses, their heterogeneity, and coupling within and between acini. Additionally, we analyzed the response to cerulein, an analogue of CCK, to demonstrate that the acinar cells in the acute pancreatic tissue slice respond to both secretagogues, and to compare their influence on active time of acinar cells. We demonstrate that confocal microscopy in conjunction with the acute pancreas tissue slice represents a viable and reliable experimental approach to evaluate both intra- and inter-cellular [Ca^2+^]_i_ signaling parameters of multiple acinar cells simultaneously, with high spatiotemporal resolution. It therefore provides a very practical platform for future high-throughput studies of normal acinar cell biology, pathophysiology, and for screening pharmacological substances.

## Materials and methods

### Animals

The tissue slices were acquired from C57BL/6J mice with the permission of the Veterinary Administration of the Republic of Slovenia (U34401-12/2015/3), following their recommendations, restrictions, and acts. The study was approved by the National Medical Ethics Committee of Slovenia (0120-369/2015-2). Mice of both sexes (6 females and one male) were kept in identical caging conditions (12:12 day to night ratio, standardized cage ventilation, standardized cages, and food) and sacrificed at 4 to 6 months with non-fasting glucose levels at 6.8 mmol/l-10.1 mmol/l.

### Chemicals and solutions

For tissue slice preparation, 1.9% low-melting-point agarose (Lonza Rockland Inc., Rockland, Maine, USA) dissolved in extracellular solution (ECS) was used. The ECS consisted of 125 mM NaCl, 26 mM NaHCO_3_, 6 mM glucose, 6 mM lactic acid, 3 mM myo-inositol, 2.5 mM KCl, 2 mM Na-pyruvate, 2 mM CaCl_2_, 1.25mM NaH_2_PO_4_, 1mM MgCl_2_ and 0.5 mM ascorbic acid, and was constantly bubbled with carbogen (v/v 95% O_2_, 5% CO_2_) to adjust pH to 7.4. The HEPES-buffered saline was composed of 150 mM NaCl, 10mM HEPES, 6 mM glucose, 5 mM KCl, 2 mM CaCl_2_, 1mM MgSO_4_, titrated to pH = 7.4 with 1 M NaOH. The dye-loading solution for confocal fluorescent imaging was composed of: 6 μM Oregon Green 488 BAPTA-1 AM Ca^2+^ dye (OGB-1 Invitrogen, Eugene, Oregon, USA) or Calbryte 520 AM Ca^2+^ dye (AAT Bioquest, Sunnyvale, California, USA), 0.03% Pluronic F-127 (w/v), and 0.12% dimethylsulfoxide (v/v), dissolved in HEPES. Stimulus solution was prepared by adding Acetylcholine chloride (ACh, ≥ 99% purity, TLC) at indicated concentrations to the ECS. All chemicals were purchased from Sigma-Aldrich (St. Louis, Missouri, USA), unless specified otherwise.

### Tissue slice preparation

**Fig 1:**
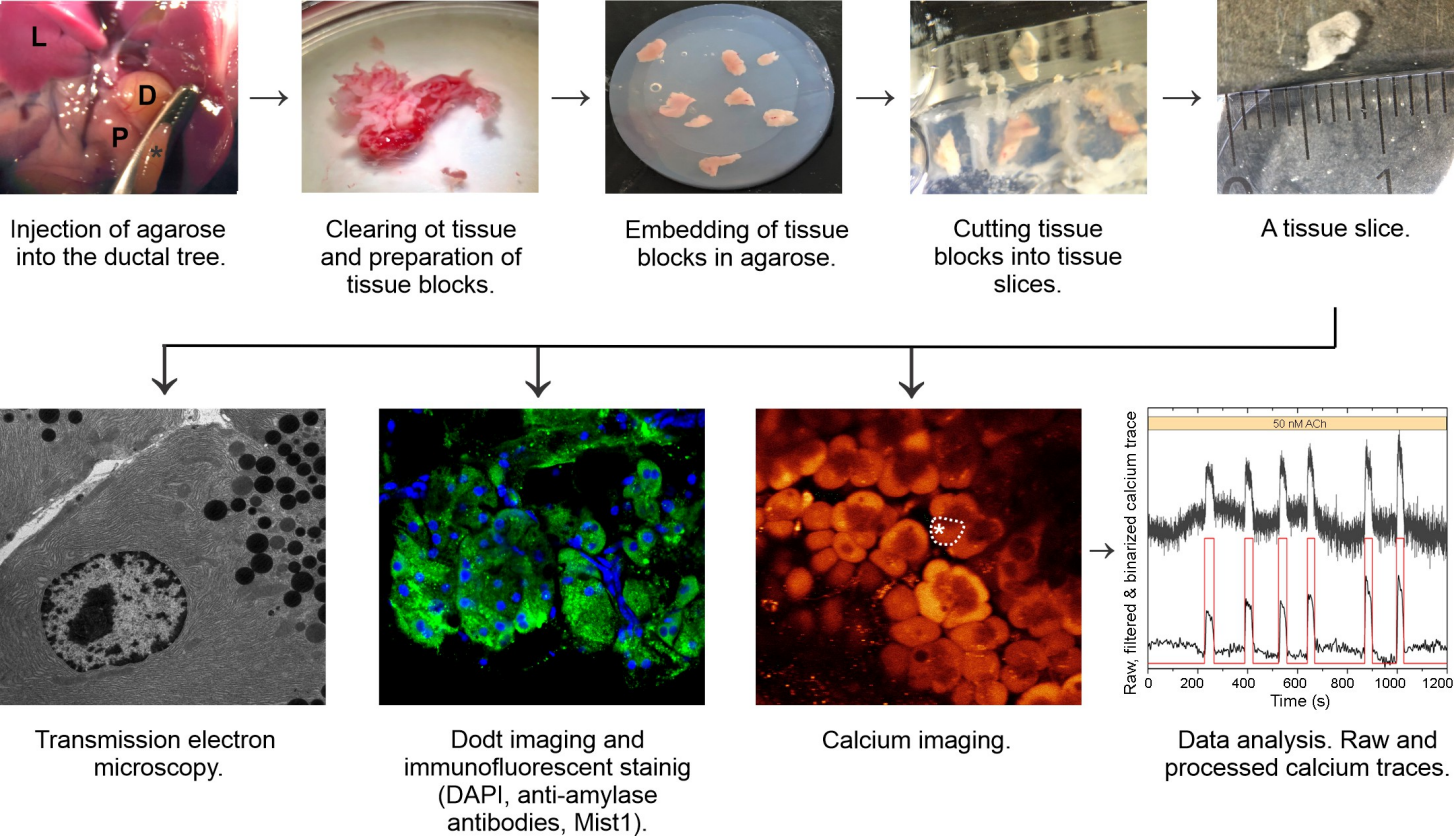
Flowchart for studying calcium responses in acinar cells in mouse pancreas tissue slices, from the injection of agarose into the ductal tree of the mouse pancreas and preparation of tissue slices to structural and functional imaging with data analysis. L, liver; P, pancreas; D, duodenum; asterisks, clamped major duodenal papilla.

Tissue slices were prepared as described in detail previously [58, 61, 70, 75]. A flowchart summarizing the main steps of the protocol is depicted in Fig 1. Briefly, following exposure to CO_2_ and cervical dislocation, laparotomy was performed and the major duodenal papilla was identified and clamped. Low-melting-point 1.9% agarose at 40°C was injected into the proximal common bile duct in order to fill the pancreas retrograde. Immediately after injection the pancreas was extracted and cooled with ice-cold ECS. We dissected the tissue to ~ 2 mm^3^ tissue blocks, removed connective and vascular tissue, and embedded it into the agarose. Blocks of agarose-injected and embedded tissue were cut at 0.08 mms^-1^ and 90 Hz to 140 μm thick slices in the ice-cold ECS solution (VT 1000 s Vibratome, Leica, Nussloch, Germany). The tissue slices were immersed into the dye-loading solutions (OGB-1 or Calbryte 520 AM) for 60 minutes on an orbital shaker (50 turns min-1) at room temperature (RT). We kept the tissue slices in HEPES solution at RT until further use for calcium imaging, electron microscopy, LIVE/DEAD assays, and immunofluorescent staining.

### LIVE/DEAD assay

To assess cell viability, the LIVE/DEAD^®^ Viability/Cytotoxicity Kit (Invitrogen, Thermo Fisher Scientific, Waltham, USA) double staining kit was used. In brief, we labeled the cells with calcein-AM and ethidium homodimer-1 (EthD-1), whereby calcein-AM stains viable cells with green fluorescence and EthD-1 stains dead cells with red fluorescence. The fluorescent dyes were excited at 488 nm using Leica TSC SP5 AOBS Tandem II upright confocal system (20x water immersion, NA 1.0) or a Leica TCS SP5 DMI6000 CS inverted confocal system (20X HC PL APO Oil, NA 0.7). Emission spectra were collected at 520 ± 20 nm for calcein-AM and 680 ± 60 nm for EthD-1 [76]. Z-stacks were obtained using the LAS AF software (Leica systems) and analyzed using Fiji.

### Transmission Electron Microscopy (TEM)

For TEM, acutely prepared tissue slices were fixed in 2.45% (v/v) glutaraldehyde and 2.45% (v/v) paraformaldehyde in 0.1 M sodium cacodylate buffer (pH = 7.4) at RT for 4 hours and at 4˚C for 16 hours. After fixation, the pieces were washed out with 0.1 M sodium cacodylate buffer (pH = 7.4) at RT for 3 hours and post-fixed with 2% OsO_4_ at RT for 2 hours. Tissue dehydration followed with step-up ethanol concentration exposure for 30 minutes (concentrations of ethanol used; 50%, 70%, 90%, 96%, and 100%) and embedded in the TAAB epoxy resin (Agar Scientific). Ultra-thin sections of 75 nm were transferred onto copper grids, stained with uranyl acetate and lead citrate, and examined using a Zeiss EM 902 transmission electron microscope.

### Amylase secretion assay

Amylase secretion measurements were carried out based on the protocol described by Marciniak et al. 2013 [59]. Briefly, each slice was incubated in 500 μl incubation buffer with or without 0.1 nM cerulein (for stimulated secretion) for 30 min at 37°C. The supernatant was removed for subsequent measurements of amylase content. The slices were then incubated in 500 μl incubation buffer containing 3% Triton-X-100 for 10 min at room temperature with 1 min thorough vortexing. The debris were centrifuged, and the supernatant was removed. Amylase content of the supernatants was measured using a commercial colorimetric kit (Diagnosticum, Budapest, Hungary) and a Biosan HiPo MPP-96 microplate photometer (Biocenter, Szeged, Hungary) [59].

### Immunofluorescent staining and imaging

The immunofluorescent staining was performed in 6-well culture plates in a batch of tissue slices separated from the ones for calcium imaging. The wells were lined with parafilm that prevented adhesion of slices to the bottom of the plate and allowed incubation in a relatively small volume. Pancreatic tissue slices were fixed in 4% (v/v) paraformaldehyde for 2 h at 4°C while shaking, followed by washing five times for 10 min with PBS containing 0.3% (v/v) Triton X-100, at RT. Slices were incubated with a blocking buffer (10% (v/v), goat serum, and 0.3% (v/v) Triton X-100 dissolved in PBS for 2 h at RT followed by immunofluorescence staining with mouse monoclonal anti-amylase antibody (1:100 dilutions, Santa Cruz, Cat. No.: sc-514229) or rabbit monoclonal Mist-1/ bHLH15 antibody (1:100 dilution, Cell Signaling Cat. No. 14896T) overnight at 4°C. Slices were afterward washed five times with PBS and incubated with the secondary antibody, Alexa Fluor 488 goat anti-mouse (1:400 dilutions, Thermo Fisher Scientific, Cat. No.: A28175), Alexa Fluor 647 goat anti-rabbit (1:500 dilutions, Thermo Fisher Scientific, Cat. No. A28175) and 4’, 6-diamidino-2-phenylindole (DAPI; 2.5 μg/ml)) for 2h at RT while shaking. Slices were then washed six times for at least 10 min with PBS and kept in PBS at 4°C in the dark until imaging. For imaging, tissue slices were fixed to the bottom of a 35 mm glass dish with an anchor. Immunofluorescent images were acquired with a Zeiss LSM 880 confocal laser scanning microscope (Carl Zeiss Technika Kft., Budaörs, Hungary) using 40 x objective lens (Plan-Apochromat 40x/1.4 oil) with a z step of 1 μm (10 confocal images @ 2048 x 2048 pixels).

### Calcium imaging

Immediately before imaging, we transferred individual tissue slices into a temperature-controlled (37°C) recording chamber of the confocal microscope. A peristaltic pump-based system perfused the recording chamber with ECS, and stimulation was performed by manually switching the system inlet between solutions. Two step-wise concentration-ramp protocols with ACh (prestimulatory 1 minute followed by 5 minutes per step) were applied: either the 5 nM, 10 nM, 25 nM, and 50 nM, spanning sub-physiological to physiological values (low protocol), or the 50 nM, 100 nM, 250 nM, 500 nM, and 1000 nM, spanning physiological to supraphysiological values (high protocol). In either case, a washout period was allowed to detect the deactivation of acinar cells. As a pilot study, we tested higher ACh concentrations of 10 μM in 2 slices and 100 μM in 4 slices. Additionally, a single cerulein protocol was used with 10 pM, 100 pM, and 1000 pM concentrations, which lies within the range of previously used concentrations [59]. A single stimulation protocol was performed per slice (ACh: 12 slices for the low protocol, 13 slices for the high protocol, both from 5 animals; cerulein: 11 slices, 2 animals).

The [Ca^2+^]_i_ dynamics were imaged with a Leica TSC SP5 AOBS Tandem II upright confocal system (20x water immersion, NA 1.0) or a Leica TCS SP5 DMI6000 CS inverted confocal system (20X HC PL APO Oil, NA 0.7) at a resolution of 5 Hz and 256 x 256 pixels or 10 Hz and 512 x 512 pixels for the higher-resolution recordings employed to detect possible apical-to-basal spreading of signal within cells. The employed Ca^2+^ dyes OGB-1 and Calbryte 520 AM were excited with the Argon 488 laser line, and emitted fluorescence was collected in the range of 500–700 nm (Leica HyD detector). Calbryte 520 AM was used to be better able to detect possible apical-to-basal differences in signals (S1 Fig) due to its resistance against extrusion from the cell, better signal to noise ratio, lower resting fluorescence with a high increase in fluorescence upon binding [Ca^2+^]_i_, and a larger mean number of events detected per cell per second compared to OGB-1 [77, 78]. Both Ca^2+^ dyes reported similar [Ca^2+^]_i_ oscillations and the type of the dye employed did not influence the results (see S2 Fig).

### Processing of calcium signals and data analyses

Regions of interest (ROIs) were selected manually off-line, based on morphological characteristics, described as a single layer of pyramidal cells concentrically distributed around the intercalate duct, and characteristic [Ca^2+^]_i_ dynamics, using the Leica LAS AF software, and exported as time series data. To identify an acinus or acinar cells in the tissue slice, scanning gradient (Dodt) contrast [79], using the 488 nm excitation laser and transmitted light detector, was used to recognize cell boundaries and apical poles (S1B Fig). Additionally, basal differences in signal intensities between cells, as well as qualitative differences in stimulated signals between acini and temporal delays between signals in individual cells from the same acinus helped distinguish individual acini and cells (S1C Fig). Further analysis of the time series data was performed using custom code in MATLAB/Python scripts (MathWorks, Inc., Massachutesetts, Germany and Python Software Foundation, Beaverton, USA). A combination of linear and exponential fitting of the data accounted for photobleaching, and the data are presented as *F*(*t*)/*F*_0_(*t*), where *F* denotes the raw signal at time point *t* and *F*_0_ the fitted baseline value at the same time point. Subsequently, the data were filtered with a zero-lag digital filter to reduce noise levels and other artefacts. Binarization of data was performed based on the onsets, peaks, and endings of oscillations. Cells that failed to exhibit sufficient signal/noise ratios to allow binarization were discarded from further analysis. The binarized data was used to calculate the parameters of the acinar response: the average frequency, the average duration, the average relative active time and the average coefficient of the inter-oscillation interval variability (*IOIV)*. Specifically, the average relative active time was determined as the fraction of 1 (i.e., “on” states) in the binarized signals, reflecting thereby the average fraction of time that cells spend in an active state with increased [Ca^2+^]_i_. The coefficient of inter-oscillation interval variability was defined as the ratio between the standard deviation of inter-oscillation interval lengths and the corresponding mean interval length [80]: *IOIV*_*i*_ = SD(*TO*_*i*_)/<*TO*_*i*_> where *TO*_*i*_ stands for the sequence of intervals between individual oscillations in the *i*-th cell. Altogether, we pooled 625 acinar cells from 214 acini in experiments with ACh and 606 acinar cells from 180 acini in experiments with cerulein. For the quantification of acinar cell synchronicity, we calculated the coactivity coefficient. This metric marks the overlap of binarized activity between different cell pairs [81]. The values of the coactivity encode the degree of cellular synchronization, whereby 0 indicates completely independent and non-overlapping activity and 1 a complete overlap between the binarized signals of a given cell pair. Coactivity coefficients between all cell pairs served also as a basis for the construction of the functional connectivity network, which features synchronous inter-cellular activity patterns [69, 81, 82].

### Statistical analyses

Statistical analyses were performed with SigmaPlot 11.0 version (Systat Software, Inc., Illinois, USA). Statistics were calculated using ANOVA on Ranks and posthoc Dunn’s method. For box-plot presentations of the data, boxes determinate the interval between the 25th and the 75th percentile, whiskers denote the minimal and the maximal values, lines within the boxes indicate the median, and small squares indicate the average value. Significant differences are indicated by asterisks (*, p<0.05; **, p<0.01; ***, p<0.001).

## Results

### Acinar cell viability and morphology in pancreas tissue slices

**Fig 2:**
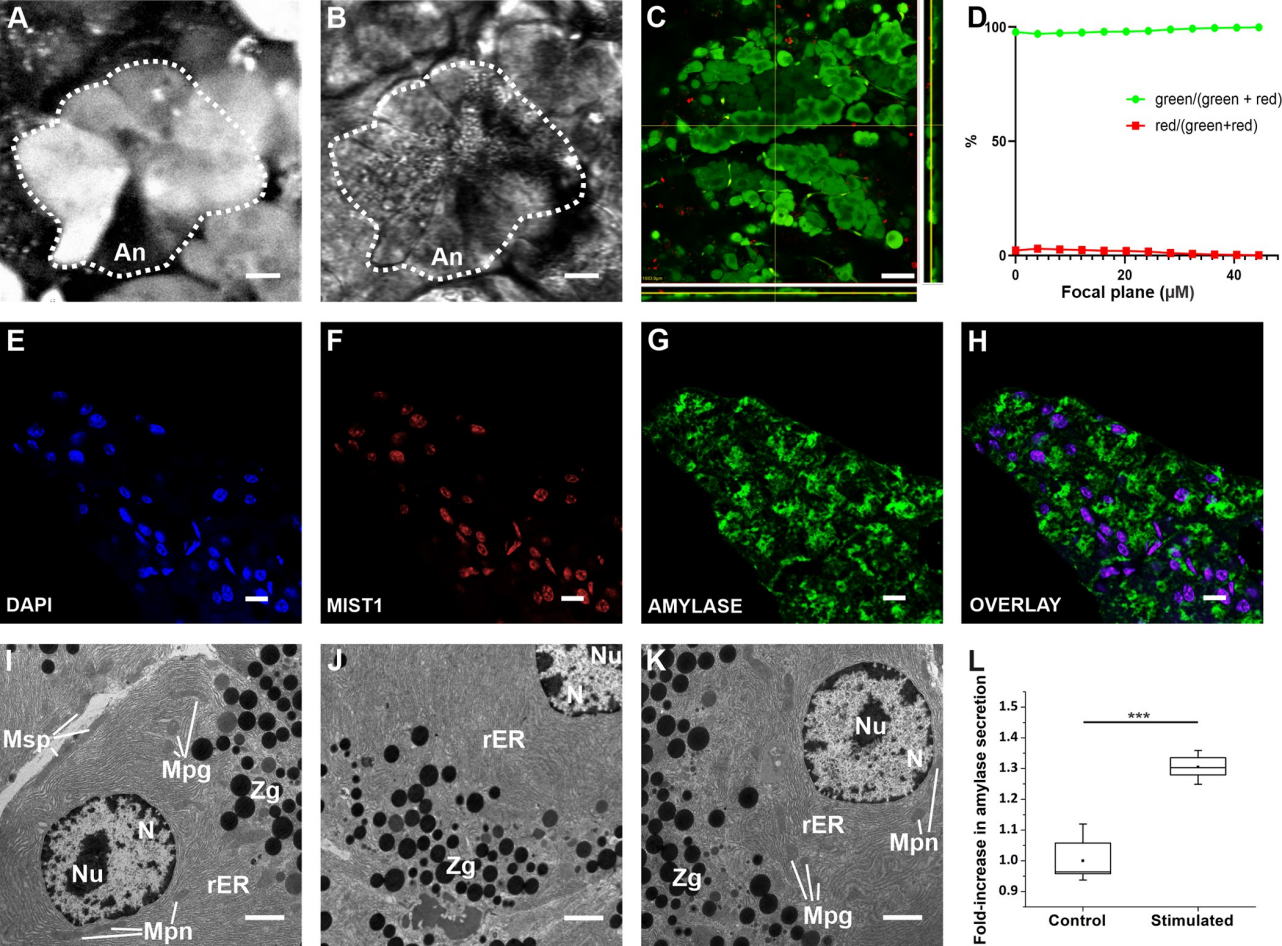
Morphology of acinar cells in mouse pancreas tissue slices. (A, B) High resolution confocal fluorescence images of the exocrine part of the pancreas, labeled with OGB-1 (A) and imaged with Dodt contrast (B). An; acinus is encircled by the broken line. Scale bar 10 μm. (C) A layer from a z-stack, cells were loaded with the Live/Dead dye. Side panels represent orthogonal projections of the z-stack, positioned as indicated by yellow lines. Pseudo color-coded, such that green indicates live cells and red indicates dead cells. Scale bar 50 μm. (D) The percentage of live cells in green and dead cells in red, as a function of depth of the focal plane within the slice. (E, F, G, H) Staining of nuclei with DAPI (E) and anti-Mist1 antibodies (F), and of cytoplasm with anti-amylase antibodies (G), with the overlay (H). Scale bar 10 μm. (I-K) TEM images of acinar cells from a tissue slice. Mpn, perinuclear mitochondria; Mpg, perigranular mitochondria; Msp, subplasmalemmal mitochondria; Zg, zymogen granules; N, nucleus; Nu, nucleolus; rER, rough endoplasmic reticulum. Scale bar 10 μm. (L) Quantification of amylase secretion upon stimulation by 0.1 nM cerulein, n = 6. Significant difference is indicted by asterisks (***, p<0.001).

To verify the viability and morphological integrity of acinar cells within acute mouse pancreas tissue slices following isolation, cutting, and loading of the dye, we performed a set of four different and complementary assessments of their structure and ultrastructure. First, high-resolution imaging of the OGB-1 or Calbryte 520 AM loaded tissue revealed pyramidal shaped acinar cells concentrically distributed around an intercalate duct or lumen, forming a typical acinus (Fig 2A and 2B). The dye localized more strongly to the basal pole of the cells, whereas the signal was less in the apical pole, consistent with the polarity of the acinar cells [13, 83]. Second, the slicing procedure hardly affected the viability of the acinar cells as the majority of the cells appeared viable and only a few cells close to the cutting surface appeared dead on the Live/Dead assay (Figs 2C and S3). Third, the ultrastructure of the acinar cells was retained during the slicing procedure. TEM revealed (i) oval-shaped mitochondria in the perinuclear, perigranular and subplasmalemmal region; they were characterized by a double membrane, the inner mitochondrial layer formed typical cristae, (ii) rough endoplasmic reticulum that was visualized as electron-dense parallel lines within the cytoplasm, (iii) zymogene granules visualized as electron-dense round structures, and (iv) nuclei with double electron-dense membrane and a nucleolus, all consistent with the typical acinar cell ultrastructure (Fig 2I–2K) [84–87]. Next, immunohistochemical staining revealed that the acinar cells abundantly expressed the enzyme amylase, the acinar cell end-product (Fig 2E, 2G and 2H) and stimulation of slices by 0.1 nM cerulein resulted in a significant increase in amylase secretion (Fig 2L), further corroborating the functional viability of acinar cells in the tissue slice [88, 89]. Finally, Fig 2F featuring immunofluorescence against the basic helix-loop-helix transcription factor Mist-1 shows that the exocrine pancreas organization and acinar cell identity were maintained in the tissue slice *in situ*, at least over the time period during which our experiments were performed.

### Dose-dependent [Ca^2+^]_i_ responses to ACh

**Fig 3:**
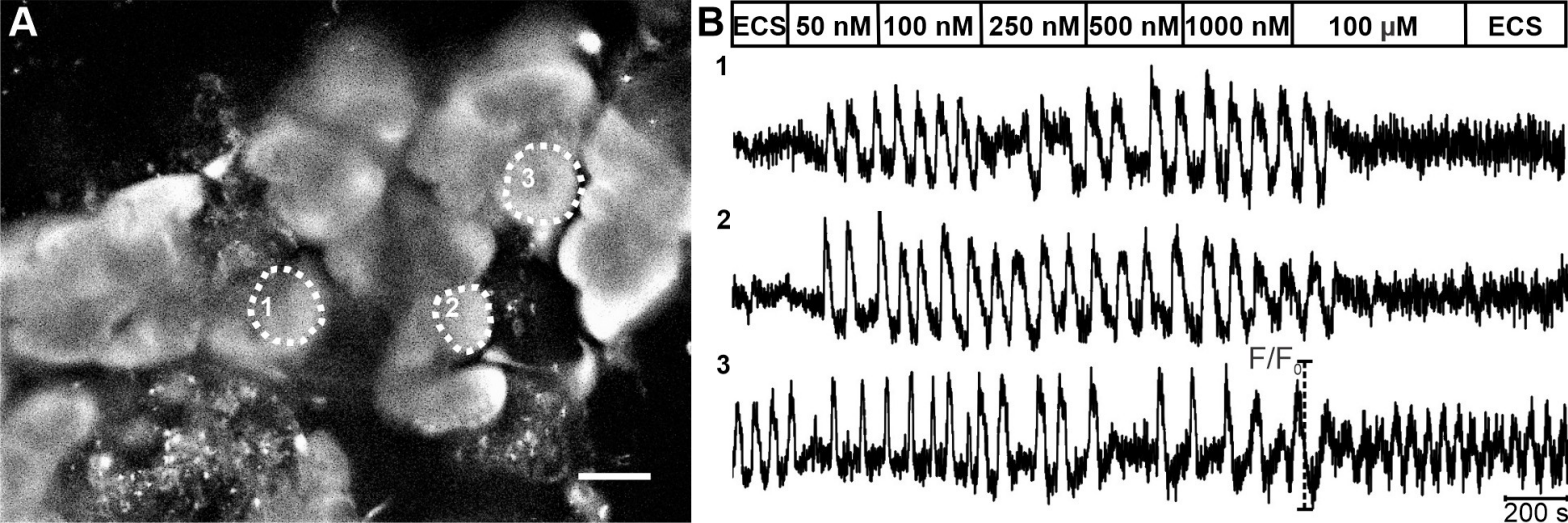
[Ca^2+^]_i_ oscillations in acinar cells upon stimulation with a ramp of ACh concentrations. (A) OGB-1 loaded acinar cells in a tissue slice. Scale bar 20 μm. (B) [Ca^2+^]_i_ oscillations shown here belong to the acinus marked with thin broken line and the corresponding number in (A). At the beginning and end of the protocol, extracellular solution (ECS) was used (see Materials and Methods). On average, after stimulation with 100 μm ACh, a biphasic [Ca^2+^]_i_ response with a rise in basal [Ca^2+^]_i_ level was seen in 98.5% of acinar cells. Note that in cell 3, spontaneous [Ca^2+^]_i_ activity was observed prior to stimulation, whereas cells 1&2 lacked spontaneous activity.

We resorted to functional multicellular confocal imaging of [Ca^2+^]_i_ dynamics of mouse acinar cells to characterize their response to Ach and cerulein in tissue slices. A pilot study indicated that in slices, concentrations > 1000 nM saturated the acinar cell response to ACh. We detected a tonic increase in [Ca^2+^]_i_ in 86% of cells (n = 43) at 10 μM and in 98% cells (n = 133) at 100 μM (Fig 3). Due to the lack of oscillatory activity at concentrations > 1000 nM, we selected two step-wise protocols composed of sequentially increasing ACh concentrations: one spanning a lower (5–50 nM) and the other a higher (50–1000 nM) concentration range. A latent period of 90–120 s was observed before cells reacted to secretagogue stimulation. In approximately half of the tested cells (54.2%, n = 339/625), no activity was detected in the non-stimulatory conditions, and the ACh stimulation provoked a response pattern consisting of oscillatory changes in [Ca^2+^]_i_. The remaining half of the tested cells expressed oscillatory activity prior to stimulation, while the ACh stimulation increased the frequency and/or duration of oscillations. Fig 3 shows spontaneous [Ca^2+^]_i_ oscillations in cell 3 and no spontaneous activity in cells 1 and 2. The response pattern was qualitatively and quantitatively similar between spontaneously active and inactive cells, and consistent with previously published data [21, 25, 90, 91].

**Fig 4:**
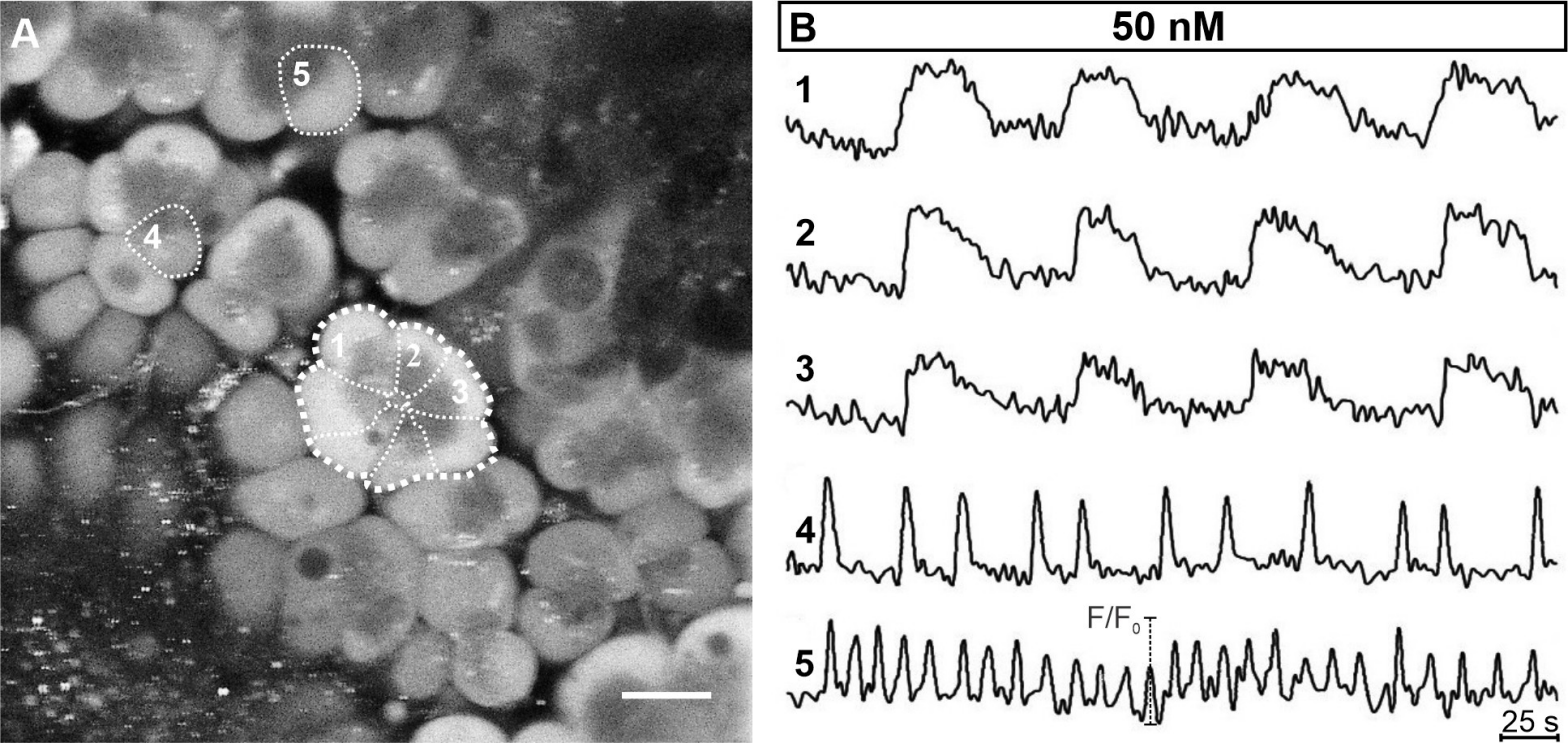
[Ca^2+^]_i_ oscillations within different acini. (A) OGB-1 loaded acinar cells in a tissue slice. Cells indicated with 1–3 belong to the same acinus (the thicker broken line marks the acinus, the thinner broken lines mark boundaries between individual acinar cells). Scale bar 20 μm. (B) The [Ca^2+^]_i_ activity patterns in three different acinar cells from the same acinus (1–3) and in two cells from two other distinct acini (cells 4 and 5) stimulated with 50 nM ACh. Please note that [Ca^2+^]_i_ oscillations were similar in shape and synchronized between cells in the same acinus but not between cells from different acini (1–3 vs. 4 vs. 5).

Fig 4 compares [Ca^2+^]_i_ oscillations within and between acini. The pattern of activity was comparable between cells belonging to the same acinus (compare cells 1–3 in Fig 4). Comparison of [Ca^2+^]_i_ patterns between acini revealed great variability in terms of both frequency and duration of oscillations (compare responses of cells 4 and 5 belonging to two separate acini, with the responses of cells 1–3 from a third acinus in Fig 4). Moreover, we detected local differences in the [Ca^2+^]_i_ increase within an acinar cell, with the signal at the apical pole preceding the signal at the basal pole by several seconds (S1 Fig), consistent with what has been reported previously [25].

**Fig 5:**
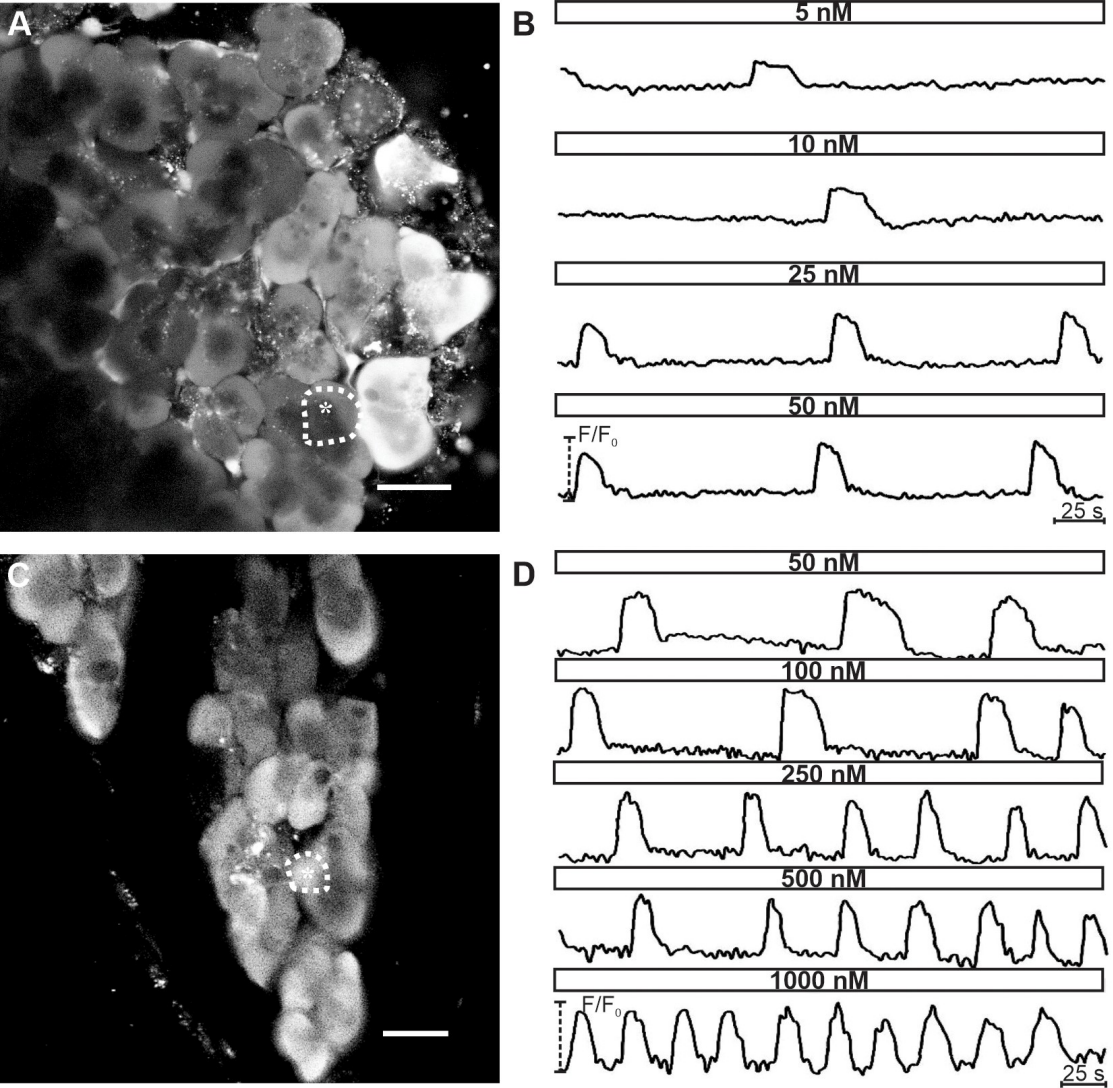
[Ca^2+^]_i_ oscillations in acinar cells stimulated with ACh display concentration-dependence. (A) and (C) OGB-1 loaded acinar cells in a tissue slice. Scale bar in Fig 5A 10 μm and in Fig 5C 20 μm. (B) [Ca^2+^]_i_ oscillations in an acinar cell in response to ACh concentrations ranging from 5 to 50 nM. The corresponding acinar cell is marked with thin broken line and asterisk in (A). (D) [Ca^2+^]_i_ oscillations cell in an acinar cell in response to ACh concentrations ranging from 50 to 1000 nM. The corresponding acinar cell is marked with thin broken line and asterisk in (C).

**Fig 6:**
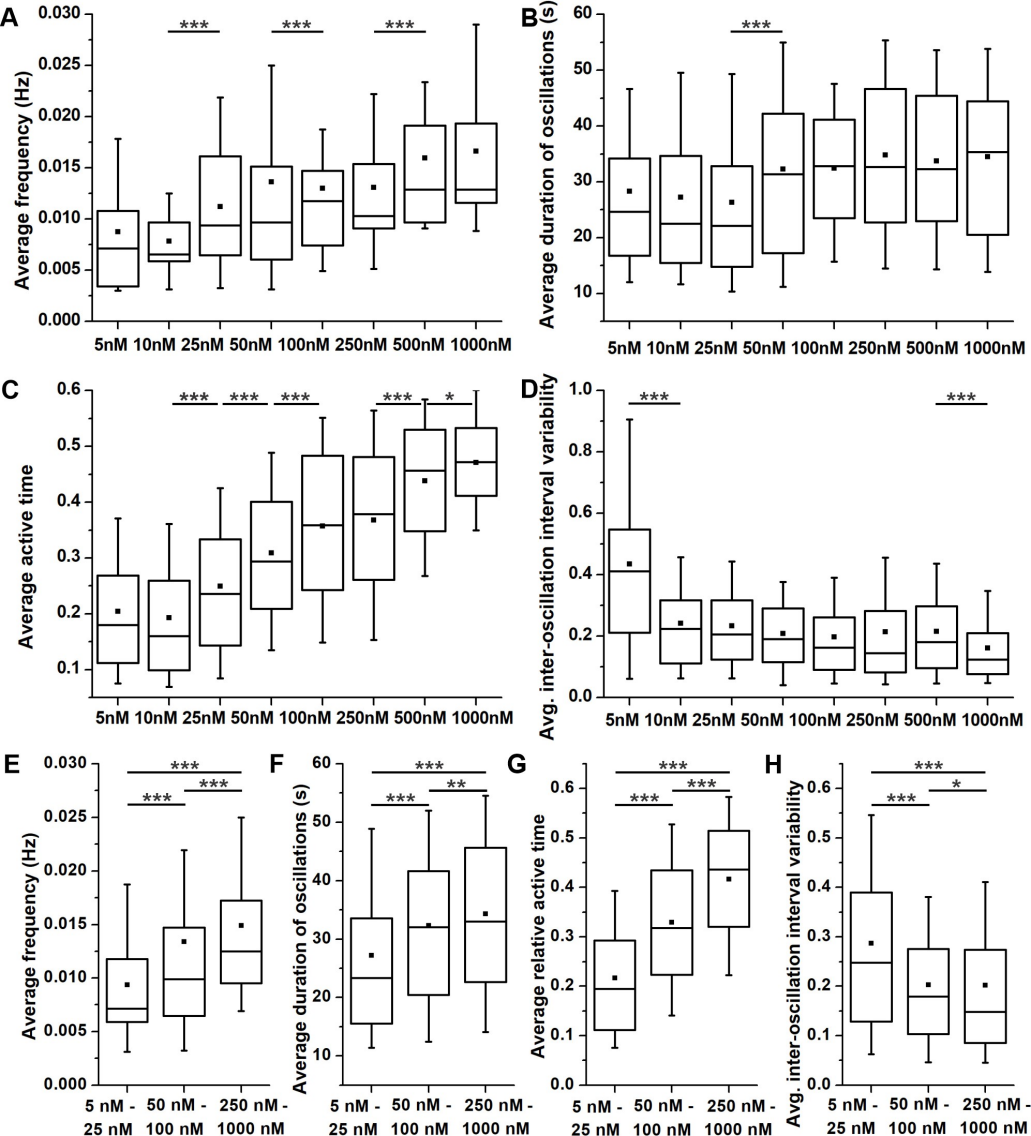
The dose-dependence of [Ca^2+^]_i_ oscillations in acinar cells upon stimulation with different concentrations of ACh. (A) and (E) Average frequency of oscillations. (B) and (F) Average duration of oscillations. (C) and (G) Average relative active time of oscillations. (D) and (H) Average inter-oscillation interval variability. Boxes determinate the interval within the 25th and the 75th percentile, whiskers denote the minimal and the maximal values, lines within the boxes indicate the median, and small squares stand for the average value. Significant differences are indicted by asterisks (*, p<0.05; **, p<0.01; ***, p<0.001). Panels E-H present pooled data for low (5–25 nM), medium (50–100 nM), and high (250–1000 nM) ACh concentrations. Number of analyzed slices/cells: 12/177 at 5 nM; 11/195 at 10 nM; 12/212 at 25 nM; 24/506 at 50 nM; 14/362 at 100 nM; 13/358 at 250 nM; 11/310 at 500 nM and 6/191cells at 1000 nM.

Furthermore, acinar cells responded to ACh stimulation dose-dependently, as exemplified in Fig 5. Increasing the concentration of ACh from 5 nM to 1000 nM gradually increased the frequency and the duration of individual oscillations. To quantify this dose-dependence, we measured the average frequency, duration, relative active time, and inter-oscillation interval variability of [Ca^2+^]_i_ oscillations (Fig 6). A significant increase in frequency and duration was observed only for some of the tested concentrations, most probably due to relatively large inter-acinar variability (Fig 6A and 6B). In an attempt to overcome the large degree of variability, we pooled the data for low (5–25 nM), intermediate (50–100 nM), and high (250–1000 nM) ACh concentration (Fig 6E and 6F). The relative increase in average oscillation frequency was larger between the low and the intermediate range (139%, median 0.007 Hz vs 0.01 Hz; p<0.001) than between the intermediate and the high range (127%; median 0.01 Hz vs. 0.013 Hz). Similarly, the average duration increased the most between the low and the intermediate range (139%; median 23.3 s vs. 32.1 s; p<0.001) and less between the intermediate and the high range (103%; median 32.1 s vs. 33 s; p<0.01). The overall activity of cells was robustly assessed by calculating the active time, presenting the fraction of time occupied by oscillations or the duty time of cells, similarly to the approach in endocrine beta cells [92]. Considering the above modulation of frequency and duration and given that, the active time is a combination of both, the average relative active time correspondingly increased with increasing ACh concentrations (Fig 6C). The highest increase in the relative active time was again observed between the low and the intermediate range of ACh concentrations (163%, median 0.195 vs. 0.318; p<0.001, Fig 6G). Finally, to evaluate the regularity of oscillations, we calculated the coefficient of inter-oscillation interval variability. A significant decrease in interval variability was observed for the most extreme of the tested concentrations (Fig 6D), and this parameter decreased more between the low and the intermediate (139%; median 0.25 vs. 0.18; p<0.001, Fig 6H) compared to the decrease between the intermediate and the high range of stimulation (120%; median 0.18 vs. 0.15; p = 0.033).

### Inter-cellular synchronization of acinar cells

**Fig 7:**
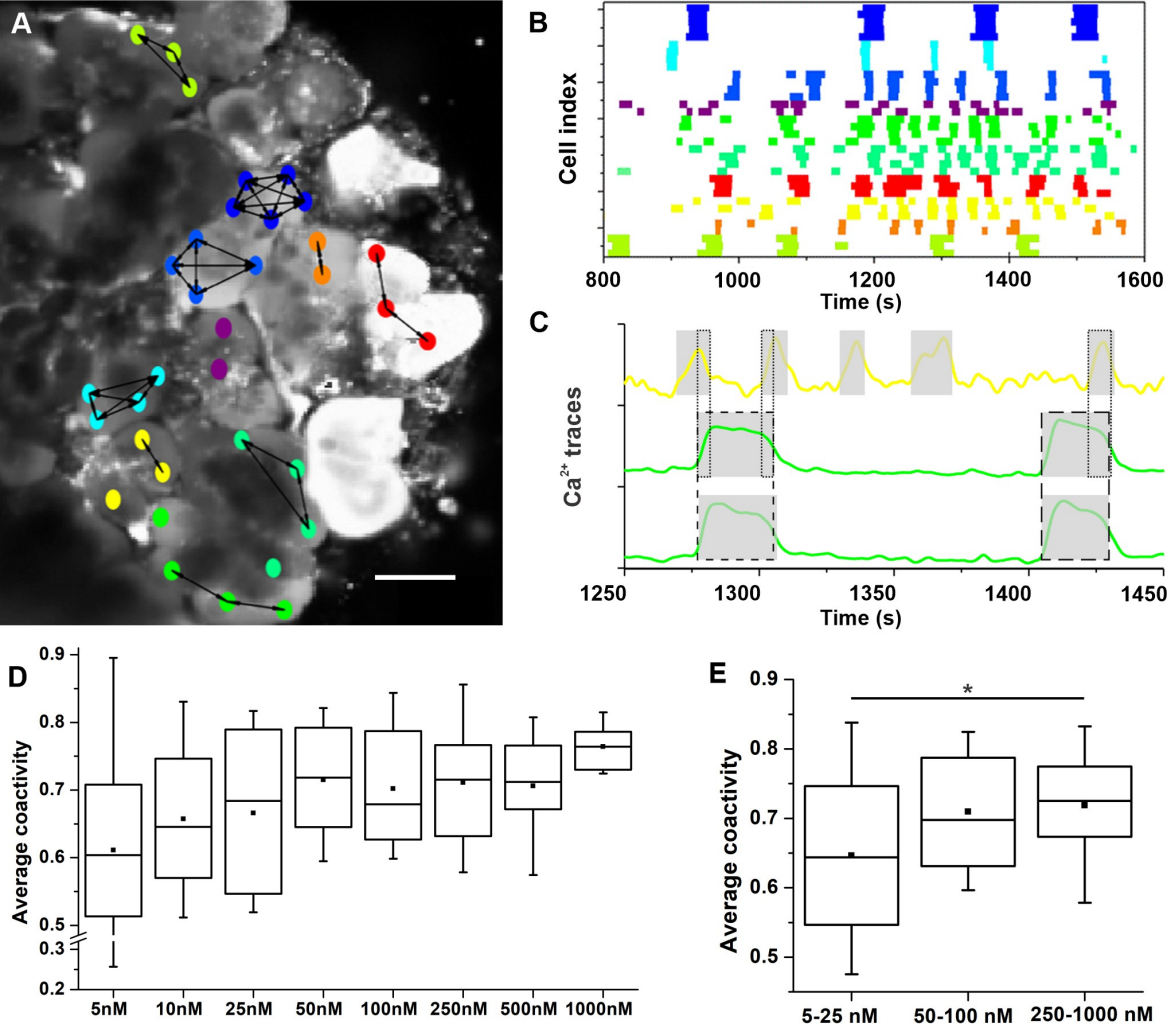
Inter-cellular synchronization of acinar cells. (A) Functional network of acinar cells extracted from coactivity and overlayed over a corresponding confocal image. Nodes denote individual acinar cells, colors code individual acini (matching the colors in panel B), and connections represent functional associations between synchronized cells, i.e., connections were established if the coactivity coefficient exceeded 0.65). Scale bar 10 μm. (B) Raster plot of binarized activity of all acinar cells in the field of view of a tissue slice during stimulation with 50 nM ACh. White color indicates no activity, other colors represent states with elevated [Ca^2+^]_i_ in particular cells, whereby colors denote individual acini. (C) Visualized coactivity between three different cells, whereby two of them were located in the same acinus (green line) and the third cell belonged to a separate acinus (yellow). The grey shaded areas indicate the duration of [Ca^2+^]_i_ oscillations of individual cells and the dashed and dotted lines the degree of coactivity (i.e., overlap in activity) between the two cells displayed in green and the middle green and yellow cell, respectively. (D) Dose-dependent average coactivity between acinar cells within individual acini. (E) Pooled data for low (5–25 nM), medium (50–100 nM), and high (250–1000 nM) ACh concentrations. Boxes determinate the interval within the 25th and the 75th percentile, whiskers denote the minimal and the maximal values, lines within the boxes indicate the median and small squares stand for the average value. Significant difference is indicted by asterisk (*, p<0.05).

As noted above, acinar cells from an acinus expressed a qualitatively more similar oscillatory pattern compared to activity in other acini (Fig 4). To quantify the level of inter-cellular synchronization, we calculated the average coactivity between pairs of cells (see Methods for detailed description and Fig 7C). First, in Fig 7A and 7B, we present a functional network of acinar cells and a raster plot of binarized activity after the tissue slice was stimulated with 50 nM ACh. Different colors of cells denote different acini. The activity was well synchronized only between cells from the same acinus, while the activity pattern differed considerably in the neighboring acini. Therefore, functional connections, reflecting well synchronized cellular activity, were established only between cell pairs within the same acini and not between the cells from the neighboring acini. Furthermore, the oscillations from the same acinus were similar also in shape (Fig 7C). Second, we computed the average coactivity coefficient to quantify the extent of synchronization within different acini and for different stimulation levels. The results showing the average intra-acinar coactivity for different ACh concentrations are presented in Fig 7D. We could not detect statistically significant differences in respect to ACh concentration, most probably due to large inter-acinar and inter-slice variability. In an attempt to overcome this issue, we again present pooled data for low (5–25 nM), intermediate (50–100 nM), and high (250–1000 nM) ACh concentration in Fig 7E, similar to Fig 6. The average coactivity coefficient between different cells within acini had a median value 0.64 in the low concentration range that increased to a significantly higher median value of 0.73 in the high concentration range.

### Dose-dependent [Ca^2+^]_i_ responses to cerulein

**Fig 8:**
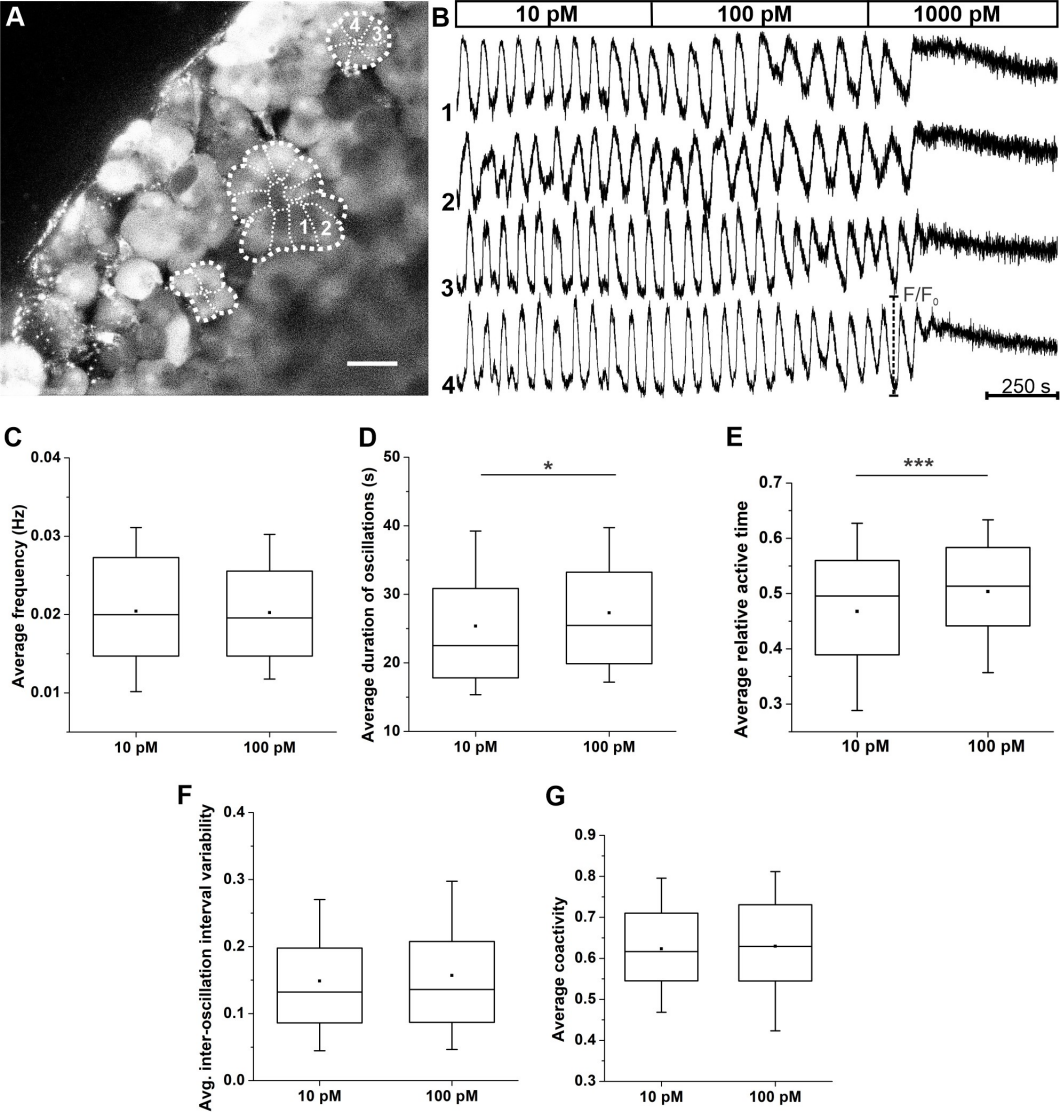
Activity of acinar cells during stimulation with cerulein. (A) Acinar cells loaded with the calcium dye. Cells indicated with 1 and 2 belong to a different acinus as cells 3 and 4 (the thicker broken line marks the acinus, the thinner broken lines mark boundaries between individual acinar cells). (B) Acinar cell activity during stimulation with increasing cerulein concentrations. Shown are four traces from two different acini, as indicated in panel A. (C) Average oscillation frequency. (D) Average oscillation duration. (E) Average relative active time. (F) Average inter-oscillation interval variability. (G) Average coactivity (G). Number of analyzed slices/cells 11/606 at 10 pM CCK and 11/571 at 100 pM CCK.

To test whether acinar cells in slices also respond to cerulein, a decapeptide cholecystokinin receptor agonist, and to compare its effect on [Ca^2+^]_i_ oscillations with responses to ACh, we stimulated slices with cerulein at a 10 pM, 100 pM and 1000 pM concentration, as employed before [59]. The results are presented in Fig 8. After applying cerulein, a latent period of 90-120s was observed before cells reacted to secretagogue and entered the phase of a stable oscillatory activity. The oscillations after simulation with 10 pM cerulein and 100 pM cerulein were the result of repetitive, semiregular cycles of elevated and subsequently decreasing [Ca^2+^]_i_ levels (Fig 8B), similarly to what was reported before [25, 91, 93, 94]. The observed oscillations were similar in the same acinus, but typically differed between acini, analogous to what we observed in the case of ACh (Fig 4). Stimulation of pancreatic acinar cells with 1000 pM cerulein evoked a biphasic increase in basal [Ca^2+^]_i_ concentration, i.e., a peak and a plateau phase, whereby the latter was characterized by a tonic and non-oscillatory increase in [Ca^2+^]_i_. A quantitative analysis revealed a dose-dependent [Ca^2+^]_i_ response to cerulein. We analyzed the activity only at 10 pM and 100 pM concentration, since the oscillations were saturated at 1000 pM (i.e., the active time amounted to unity or 100% in this case). The oscillatory activity at these two cerulein concentrations was rather high and comparable to responses detected at 500 nM or 1000 nM ACh (Fig 6). Moreover, we observed a significant increase of the duration of oscillations and relative active time at higher cerulein concentrations (Fig 8D and 8E). In contrast, the frequency of oscillations (Fig 8C), their regularity (Fig 8F), and the degree of inter-cellular synchronization (Fig 8G) were not found to be concentration-dependent.

## Discussion

Understanding normal and pathological pancreas morphology and physiology is important due to diseases that affect this bifunctional gland. The tissue slice approach has established itself as an instrumental experimental approach in the past two decades, representing a cornerstone of studies on the structure and function of endocrine [58, 62–64, 70], ductal [65], and acinar cells [50, 59, 66, 67, 74, 95]. In our previous work, we have shown that the tissue slice methodology does not critically alter the morphology and function of the islets of Langerhans and ductal cells [61, 65, 70, 96]. By using a combination of morphological and functional assessments, including confocal live-cell [Ca^2+^]_i_ imaging in the present study, we further demonstrate that the acinar cells also remain morphologically and functionally intact in tissue slices, making them an attractive future approach to study acinar cells and their association with ductal and endocrine cells. More specifically, our morphological assessments show that the viability, structure, and ultrastructure of acinar cells and their organization into acini remain largely intact in acute tissue slices, corroborating the few previous reports in mouse [50, 59] and human tissue slices [66, 67, 97].

From the functional point of view, ACh and CCK are potent triggers for the stimulus secretion cascade (SSC) that results in secretion of enzymes. Previous studies demonstrated that acinar cells respond to these agonists with [Ca^2+^]_i_ oscillations, but with different temporal patterns [91, 98, 99], that there is typically a delay between the [Ca^2+^]_i_ increase in the apical and the basal pole (S1 Fig) [25], and that high concentrations induce a biphasic response lacking oscillations [90, 100–102]. Scarce and partially conflicting data are available on how acinar cells transduce differences in ACh and CCK concentration into differences in [Ca^2+^]_i_. A threshold concentration of 1 μM ACh and 1 nM CCK induced an oscillatory response [73, 93, 103], while others reported a biphasic response even at concentrations below 1 μM or 1 nM [91, 99, 104]. Since only a few studies reported a dose-dependent change in either frequency or duration of oscillations, we understand the ACh and CCK encoding only partially [73, 91, 99]. Other indices of the coding properties came from the optical study of zymogen granule dynamics, demonstrating two distinct pools of vesicles [105]. A threshold ACh concentration of 10–50 nM activated a subpopulation of vesicles with high fusion rates, followed by recruitment of slowly releasable granules, consistent with the biphasic profile of enzyme secretion from acinar cells [12, 105–108]. While above-threshold ACh concentrations (250 nM—10 μM) increased the overall vesicle fusion rate in a dose-dependent manner, the effect was limited to the slowly releasable pool. In sum, it seems that the ACh response is modulated in at least two steps along the SCC cascade, changing both the calcium dynamics and vesicle recruitment independently. However, this view is further complicated by the oscillatory nature of the calcium response to ACh that was neglected by studies on granule dynamics.

The previous inconsistent data on the acinar cell response to different ACh concentrations were the primary motivation for our study. In our hands, acinar cells responded to ACh stimulation with repetitive [Ca^2+^]_i_ oscillations up to 1000 nM (Figs 3 and 5) and for the CCK receptor agonist cerulein up to 100 pM (Fig 8B). Applying a stimulation protocol consisting of a step-wise increase in ACh concentration from physiological to supraphysiological concentrations, we demonstrated that an increase in ACh stimulation increased both the frequency and the duration of the oscillations (Fig 6). We observed a 139% increase in both the duration and the frequency in the intermediate ACh concentration range (up to 100 nM), indicating that the coding of ACh concentrations employs both parameters equally. Higher ACh concentrations affected the acinar cells less (3–27%, Fig 6), consistent with a saturation of ACh response. Others reported that ACh, used in the range of 10–500 nM, evoked similar durations [73, 91, 99, 109], but surprisingly high frequencies (0.05–0.13 Hz), a whole order of magnitude faster than in our hands [90, 91, 94], a finding that deserves to be addressed in future studies. In experiments with cerulein, we observed an increase in duration and relative active time, when comparing physiological (10 pM) and submaximal (100 pM) concentrations. The calculated frequency and duration were 0.02 Hz and 23 s at lower cerulein values, which is consistent with previous reports [91, 93, 99, 109]. Furthermore, Tsunoda et al. reported a decrease in frequency and amplitude with higher CCK values although the peak of [Ca^2+^]_i_ was observed at 1 nM CCK, which is also consistent with our findings. This observed decrease is probably related to the large initial increase in [Ca^2+^]_i_ after low CCK stimulations [93].

At least two alternative ACh-coding mechanisms were proposed in other tissues. On the one hand, ACh-dependent recruitment of oscillating cells was reported in airway smooth muscle cells [110, 111] and in pancreatic beta cells [112]. CCK-dependent recruitment of oscillating cells was also reported in pancreatic acinar cells in terms of receptor-mediated [Ca^2+^]_i_ mobilization [113]. On the other hand, amplitude modulation was observed in cortical neurons [114], and this mechanism is most probably not involved in acinar cells [105].

Spontaneous [Ca^2+^]_i_ oscillations were observed in our study in about half of the cells. It has been reported that [Ca^2+^]_i_ oscillations in non-excitable cells, such as pancreatic acinar cells, can be preceded by a gradual increase in [Ca^2+^]_i_ that resembles a pacemaker potential. This phenomenon was first observed in endothelial cells and hepatocytes [115–117], and later, in mesenchymal stem cells [118, 119] and salivary gland ductal cells [120], and is believed to be linked to IP_3_R activation and Ca^2+^ influx, as well as an ATP-mediated autocrine/paracrine signaling pathway [121–124]. The spontaneous oscillations clearly require further attention in future studies to explain their mechanistic substrate. Since they have been described in other tissues and were present immediately at the beginning of the recording in our study, they probably represent a true biological phenomenon and were not caused by phototoxicity or excess laser exposure of cells loaded with fluorophores [125].

The [Ca^2+^]_i_ increases generated through agonist stimulation are not exclusively limited to a single acinar cell but can also propagate from one cell to another. Inter-cellular coupling between acinar cells by gap junctions consisting of Cx32 and Cx26 makes it possible for the Ca^2+^ signals to travel in a wave-like manner between the cells and thereby serve as a means of inter-cellular communication [30]. This leads to a rather coordinated and higher [Ca^2+^]_i_ activity of acinar cells when compared to isolated cells and has been suggested to affect digestive enzyme secretion [24, 29]. Alternatively, signal transmission can be facilitated by ATP release into the extracellular space or stretch-activated Ca^2+^ channels [126]. Moreover, it has been reported that stimulation with high ACh concentrations or other cholinergic analogs leads to a partial uncoupling of pancreatic acinar cells. The non-trivial modulation of gap junctional coupling between acinar cells is thought to be a crucial determinant of initiating, maintaining, or enhancing the increased secretion of pancreatic enzymes [26, 127]. Our analysis did not observe a drop in coordinated Ca^2+^ activity at the highest employed stimulation levels but a slight increase in synchronicity. However, it should be noted that our evaluation of synchronous behavior in acinar cells *in situ* does not directly reflect the extent of gap-junctional coupling only but also encompasses the overall activity and regularity of oscillations, both of which seem to increase with increasing levels of ACh (Fig 6), which was not the case for cerulein (Fig 8). Particularly under low concentrations of agonists, there appears to be a higher degree of fluctuations in oscillation periods, as under these circumstances, the [Ca^2+^]_i_ activity is more prone to stochasticity due to random opening and closing of Ca^2+^ channels [74, 128, 129]. Furthermore, our results coincide with previous reports showing that the acinar cells are well connected within acini and not between acini. More specifically, in functional cellular networks constructed from [Ca^2+^]_i_ activity, a well-stablished methodology to evaluate inter-cellular connectivity patterns [82], connections were established solely between cells from the same acini (Fig 7A). Therefore, in the future, such a functional assessment could in principle be used to help discriminate acinar cells and different acini within tissue slices and supplement the characterization based on morphological data [130, 131]. Finally, despite the rather well-aligned [Ca^2+^]_i_ oscillations within individual acini, the patterns of inter-cellular activity appear to be quite erratic and without a clear course. This is most probably related to the functional and morphological polarity, stochastic influences, and various [Ca^2+^]_i_ handling mechanisms in acinar cells. Namely, computational models that have been developed to elucidate the processes governing the inter-cellular [Ca^2+^]_i_ dynamics have shown that spatially distributed diffusion and cell geometry both play important roles in determining behavior in pancreatic acinar cells [132, 133]. Most importantly, this also strongly impacts inter-cellular activity and synchronization of oscillations between cells and results in a variety of synchronous, phase-locked, or even asynchronous behaviors [134], similar to what we observed in our experiments. To investigate these issues in further detail, we encourage additional experimental studies as well as the development of multicellular models that would incorporate acinar cell structure and heterogeneity along with the temporal and agonist-dependent variability of gap-junctional conductance. This would enable a more holistic assessment of the complex and variable [Ca^2+^]_i_ activity patterns in pancreatic acini.

To conclude, animal models of disease in combination with the acute pancreas tissue slice have been used to assess morphological changes and elucidate the background etiopathogenesis of common pancreatic disorders such as type 2 diabetes in chemically induced [64] or genetic models [135]. This methodology offers background for the use of more contemporary models of disease like Western diet induced type 2 diabetes [136] and acute pancreatitis [137] with the tissue slice technique. With the adaptation of the tissue slice technique for human and porcine tissue [50, 138, 139], findings are more suitable for application to human (patho)physiology, and the disadvantages of animal experiments can be omitted. Finally, inspiring approaches, such as the syncollin-pHluorin construct delivered by adenovirus transfection [140], and extracellular dyes that transiently enter the vesicles while in the fused state [141–143], would allow the study of spatiotemporal vesicle dynamics in a large number of acinar cells simultaneously. This approach could help explain whether calcium dynamics suffice to elucidate the vesicle fusion properties [67, 144] in health and disease and help pinpoint novel pharmacological targets [35, 145].

## Supporting information

S1 Fig [Ca^2+^]_i_ activity within a single acinar cell. (A) Acinar cells loaded with the calcium reporter dye. (B) Morphological visualization of an acinus using scanning gradient contrast (Dodt) imaging. Cell boundaries and temporal differences in signals between cells visible on calcium imaging (A), and cell boundaries and granules on apical poles visible on Dodt imaging (B) were used to identify an acinus and individual cells. Scale bar 10 μm. (C) [Ca^2+^]_i_ activity of acinar cells that were identified from calcium imaging (A) and Dodt imaging (B) to have a common orientation of the apical poles. Acinar cells are depicted and numbered. The blue and red dot indicate parts of the basal and apical pole, referred to in (E). (D) [Ca^2+^]_i_ oscillatory activity in numbered acinar cells after stimulation with 500 nM ACh. (E) [Ca^2+^]_i_ oscillatory activity from apical (blue) and basal (red) pole of the acinar cell (C) during stimulation with 500 nM ACh. Note the apical-to-basal temporal delay in [Ca^2+^]_i_ increase.
(TIF)

S2 Fig Comparison between fluorescent [Ca^2+^]_i_ indicators Oregon Green BAPTA-1 (OGB-1) and Calbryte 520 AM. [Ca^2+^]_i_ oscillations after stimulation with 100nM ACh loaded with OGB-1 (A) and Calbryte 520 AM (B). Comparison between the measured relative active times (C) and the average inter-oscillation interval variability (D) using different dyes.
(TIF)

S3 Fig Quantification of cell viability in the tissue slice preparation. A Montage of a z-stack following LIVE/DEAD double staining. Individual panels depict different focal plains (indicated with numbers). Green color indicates live cells labeled with calcein-AM and red color indicates dead cells labeled with EthD-1. Scale bar 50 μm.
(TIF)
