# Supplementary figures and images for: Calcium imaging in intact mouse acinar cells in acute pancreas tissue slices
Source: PLoS One. 2022 Jun 3;17(6):e0268644. doi: 10.1371/journal.pone.0268644 (PMC9165796; doi:10.1371/journal.pone.0268644)

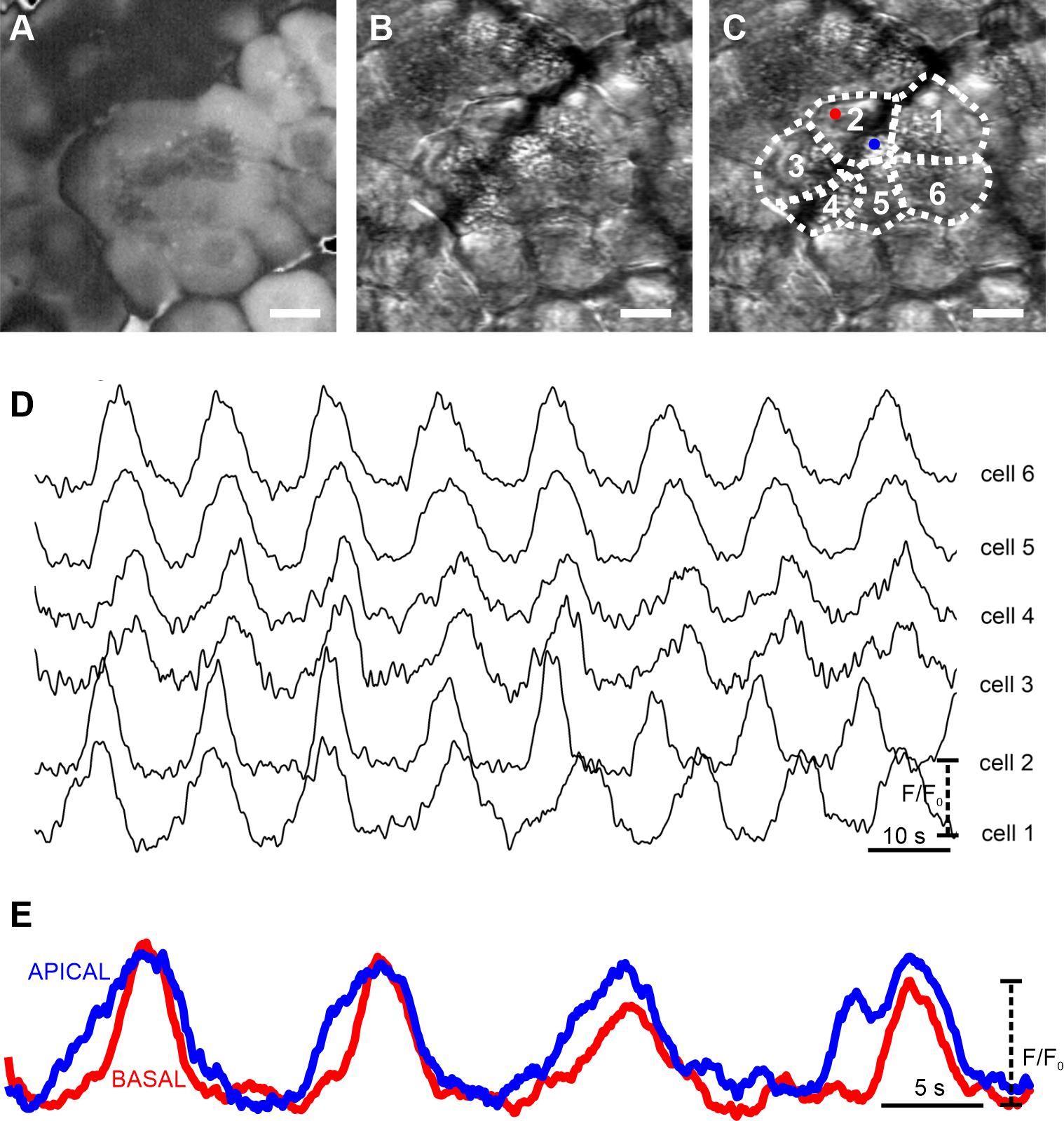

Supplement: S1 Fig — (A) Acinar cells loaded with the calcium reporter dye. (B) Morphological visualization of an acinus using scanning gradient contrast (Dodt) imaging. Cell boundaries and temporal differences in signals between cells visible on calcium imaging (A), and cell boundaries and granules on apical poles visible on Dodt imaging (B) were used to identify an acinus and individual cells. Scale bar 10 μm. (C) [Ca2+]i activity of acinar cells that were identified from calcium imaging (A) and Dodt imaging (B) to have a common orientation of the apical poles. Acinar cells are depicted and numbered. The blue and red dot indicate parts of the basal and apical pole, referred to in (E). (D) [Ca2+]i oscillatory activity in numbered acinar cells after stimulation with 500 nM ACh. (E) [Ca2+]i oscillatory activity from apical (blue) and basal (red) pole of the acinar cell (C) during stimulation with 500 nM ACh. Note the apical-to-basal temporal delay in [Ca2+]i increase. (TIF) [file pone.0268644.s001.tif]

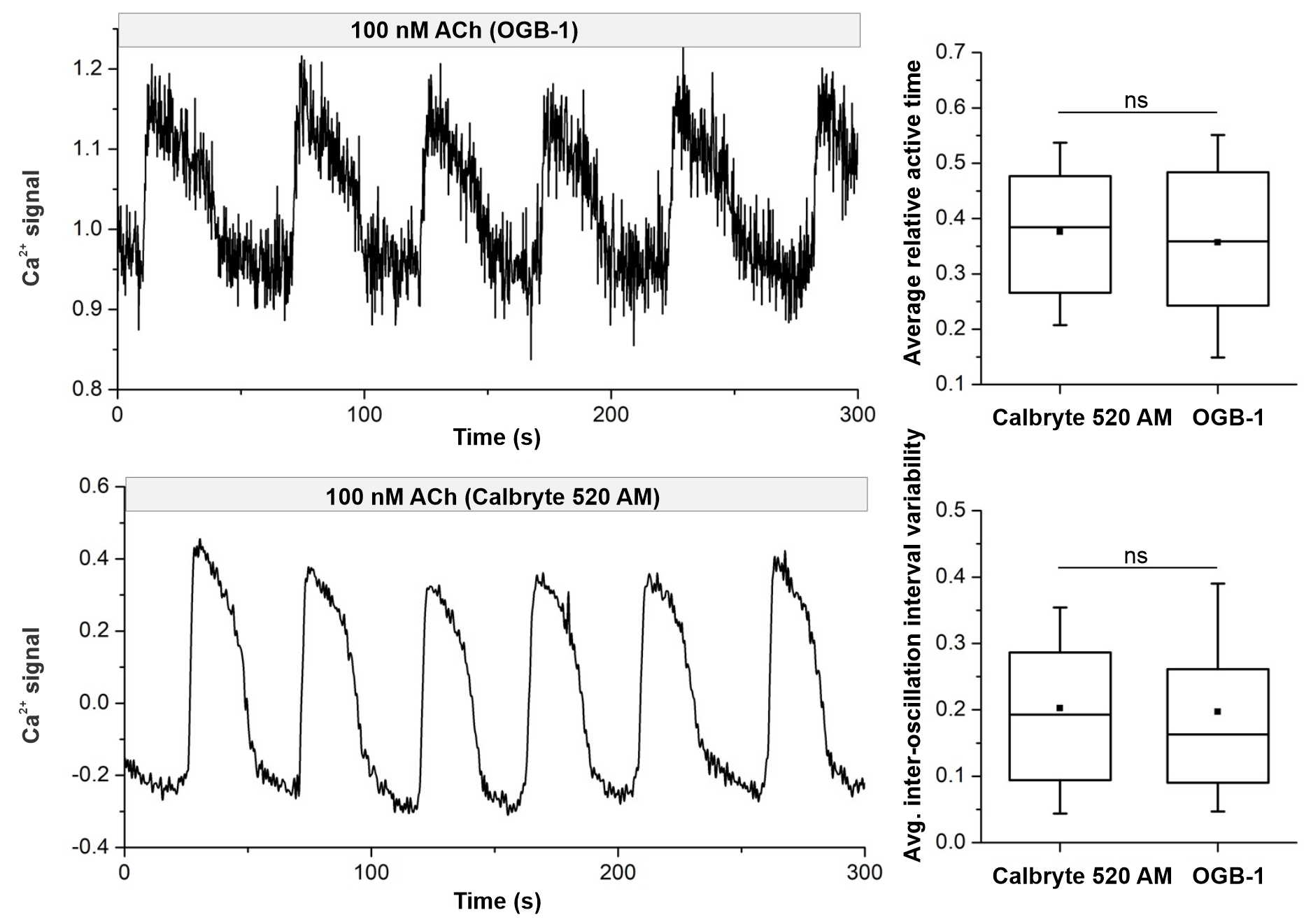

Supplement: S2 Fig — [Ca2+]i oscillations after stimulation with 100nM ACh loaded with OGB-1 (A) and Calbryte 520 AM (B). Comparison between the measured relative active times (C) and the average inter-oscillation interval variability (D) using different dyes. (TIF) [file pone.0268644.s002.tif]

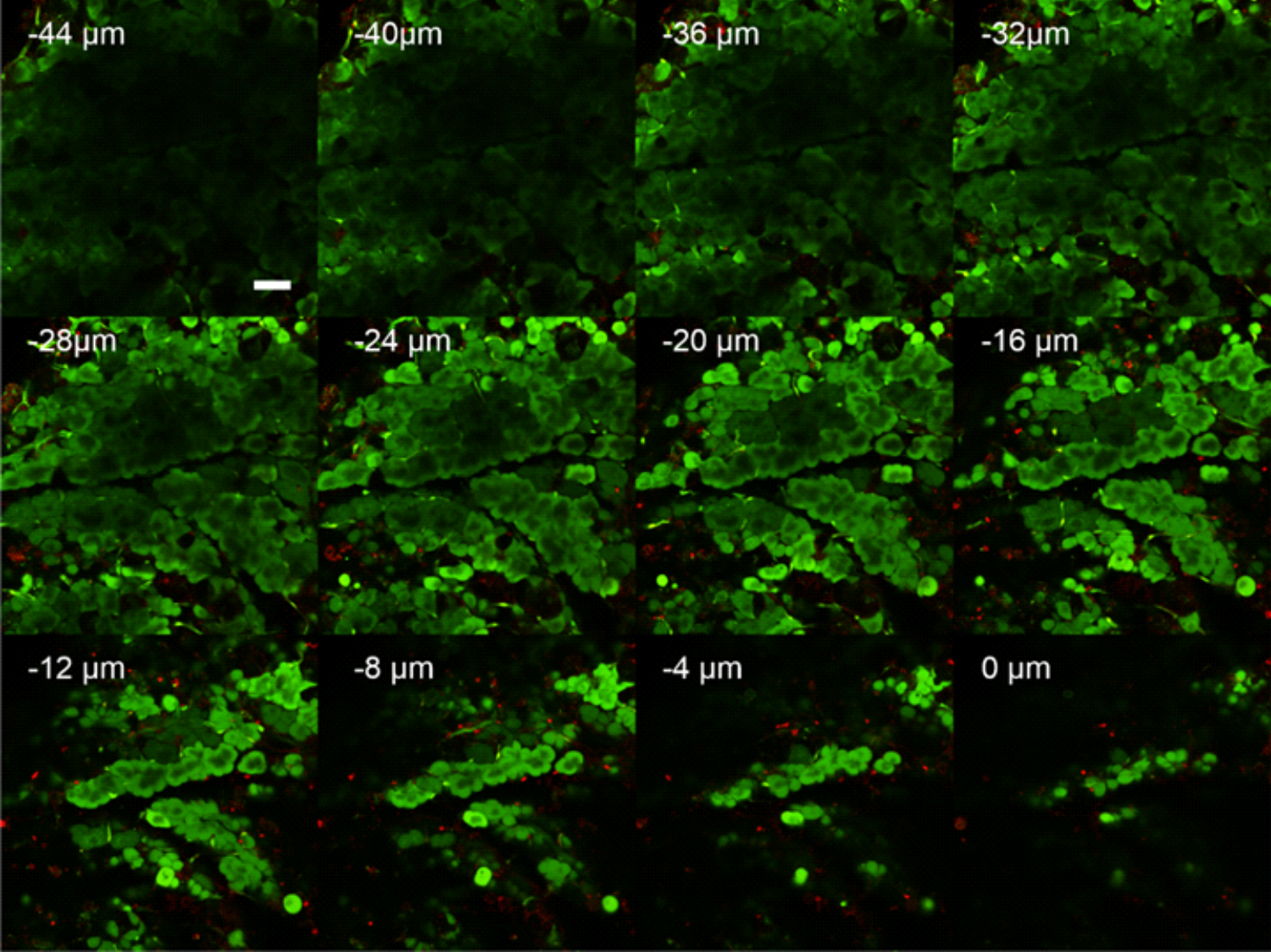

Supplement: S3 Fig — A Montage of a z-stack following LIVE/DEAD double staining. Individual panels depict different focal plains (indicated with numbers). Green color indicates live cells labeled with calcein-AM and red color indicates dead cells labeled with EthD-1. Scale bar 50 μm. (TIF) [file pone.0268644.s003.tif]
